# Ghrelin cell–expressed insulin receptors mediate meal- and obesity-induced declines in plasma ghrelin

**DOI:** 10.1172/jci.insight.146983

**Published:** 2021-09-02

**Authors:** Kripa Shankar, Shota Takemi, Deepali Gupta, Salil Varshney, Bharath K. Mani, Sherri Osborne-Lawrence, Nathan P. Metzger, Corine P. Richard, Eric D. Berglund, Jeffrey M. Zigman

**Affiliations:** 1 Center for Hypothalamic Research, Department of Internal Medicine, UT Southwestern Medical Center, Dallas, Texas, USA.; 2 Area of Regulatory Biology, Division of Life Science, Graduate School of Science and Engineering, Saitama University, Sakuraku, Saitama, Japan.; 3 Division of Endocrinology, Department of Internal Medicine, and; 4 Department of Psychiatry, UT Southwestern Medical Center, Dallas, Texas, USA.

**Keywords:** Endocrinology, Metabolism, Insulin, Mouse models, Obesity

## Abstract

Mechanisms underlying postprandial and obesity-associated plasma ghrelin reductions are incompletely understood. Here, using ghrelin cell–selective insulin receptor–KO (GhIRKO) mice, we tested the impact of insulin, acting via ghrelin cell–expressed insulin receptors (IRs), to suppress ghrelin secretion. Insulin reduced ghrelin secretion from cultured gastric mucosal cells of control mice but not from those of GhIRKO mice. Acute insulin challenge and insulin infusion during both hyperinsulinemic-hypoglycemic clamps and hyperinsulinemic-euglycemic clamps lowered plasma ghrelin in control mice but not GhIRKO mice. Thus, ghrelin cell–expressed IRs are required for insulin-mediated reductions in plasma ghrelin. Furthermore, interventions that naturally raise insulin (glucose gavage, refeeding following fasting, and chronic high-fat diet) also lowered plasma ghrelin only in control mice — not GhIRKO mice. Thus, meal- and obesity-associated increases in insulin, acting via ghrelin cell–expressed IRs, represent a major, direct negative modulator of ghrelin secretion in vivo, as opposed to ingested or metabolized macronutrients. Refed GhIRKO mice exhibited reduced plasma insulin, highlighting ghrelin’s actions to inhibit insulin release via a feedback loop. Moreover, GhIRKO mice required reduced glucose infusion rates during hyperinsulinemic-hypoglycemic clamps, suggesting that suppressed ghrelin release resulting from direct insulin action on ghrelin cells usually limits ghrelin’s full potential to protect against insulin-induced hypoglycemia.

## Introduction

Ghrelin is produced mainly by a population of gastric enteroendocrine ghrelin cells in adults (1, 2). Not only is ghrelin a growth hormone (GH) secretagogue, but it also helps respond to changes in the metabolic state. Plasma ghrelin rises with caloric restriction and is negatively correlated with body weight and adiposity (3–6). Administered ghrelin and/or ghrelin receptor (growth hormone secretagogue receptor [GHSR]) agonists stimulate food intake and food reward behavior, lower energy expenditure, and induce body weight gain (7–9). In contrast, some studies demonstrate that limiting ghrelin action reduces baseline food intake, fasting-induced refeeding, feeding responses after exercise, hedonic eating, body weight gain, and adiposity (8, 10–15). For example, administration of the endogenous GHSR antagonist/inverse agonist LEAP2 reduces baseline food intake and ghrelin-induced food intake (16, 17).

Ghrelin also has several glucoregulatory actions, including reducing insulin sensitivity and restricting insulin secretion, not to mention potentiating GH release and food intake, which contribute to its overall effect to raise blood glucose (18, 19). In contrast, pharmacological GHSR blockade improves hyperglycemia, glucose tolerance, and insulin sensitivity in diet-induced obese mice, Zucker diabetic fatty rats, and/or MODY-3 diabetic mice (20–22). Both ghrelin-KO and GHSR-KO mice require higher glucose infusion rates during hyperinsulinemic-hypoglycemic clamps to sustain the goal glycemic range (23–25). Also, ghrelin-KO mice exhibit a progressive decline in fasting blood glucose to the point of near-death following a week-long 60% caloric restriction regimen that depletes body fat to < 2% (26).

While plasma ghrelin elevations associated with caloric restriction depend largely on sympathetic nervous system engagement of ghrelin cell–expressed β1-adrenergic receptors (β1ARs) (27), molecular mediators underlying the ghrelin reductions associated with food intake and diet-induced obesity remain insufficiently characterized. Several clues suggest direct roles for ingested or metabolized macronutrients in suppressing ghrelin secretion. Not only do lipid gavage and lipid beverage ingestion decrease plasma ghrelin, but also, the short-chain and long-chain fatty acid receptors GPR43 and GPR120 are enriched within the ghrelin cell transcriptome, and both inhibit ghrelin secretion when activated by selective ligands (28–32). Ingestion of protein beverages and exposure of gastric tissue segments, isolated stomachs, or ghrelinoma cell lines to specific amino acids and amino acid receptor agonists suppress ghrelin secretion (32–34). Also, oral and parenteral glucose administration reduce plasma ghrelin (35–39). This effect of glucose may be direct, as evidenced by ex vivo studies using a mouse gastric mucosal cell primary culture model, in which medium containing high glucose inhibits ghrelin secretion (35, 40).

Other clues suggest that postprandial and obesity-associated falls in plasma ghrelin result from increased plasma insulin. Notably, preprandial and postprandial plasma insulin and ghrelin are inversely related (4, 32). While postprandial suppression of plasma ghrelin is intact in streptozotocin-treated rats that lack the usual postprandial rise in insulin, it is shortened in duration (41). Also, reductions in plasma ghrelin occur during hyperinsulinemic-euglycemic and hyperinsulinemic-hypoglycemic clamp procedures and insulin tolerance testing (24, 25, 39, 42, 43). Moreover, insulin can restrict ghrelin secretion from cultured gastric mucosal cells, presumably via direct interaction with insulin receptors (IRs), which are highly expressed in ghrelin cells (40, 44). We designed the current study to ascertain the role of ghrelin cell–expressed IRs on meal- and obesity-induced reductions in plasma ghrelin and the physiological impact of disrupting that signaling.

## Results

### Generation and validation of GhIRKO mice.

**Figure 1:**
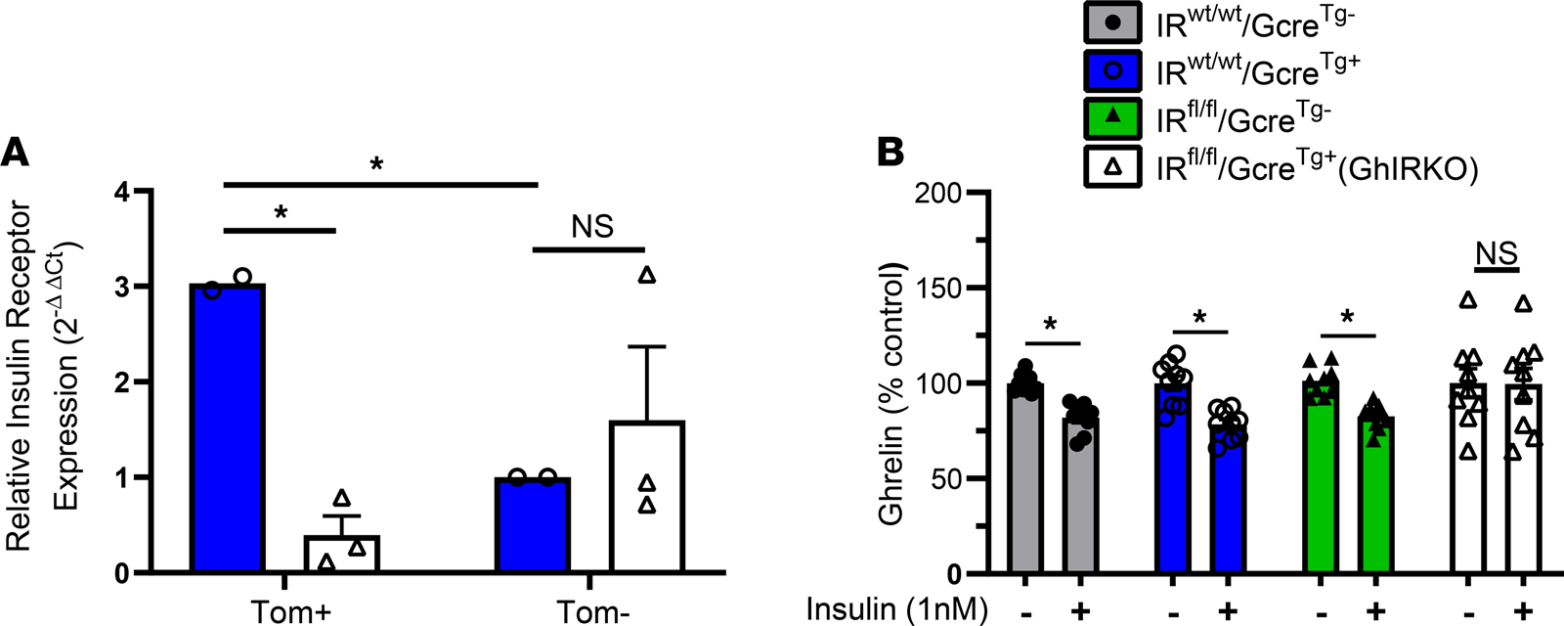
Validation of ghrelin cell–selective deletion of IR gene expression in GhIRKO mice. (**A**) IR mRNA expression in FACS-purified gastric mucosal ghrelin cells (Tom^+^) and nonghrelin gastric mucosal cells (Tom^–^) obtained from IR^fl/fl^/GCre^Tg+^ (GhIRKO) mice and IR^wt/wt^/GCre^Tg+^ control mice on a tdTomato reporter background, as determined by qPCR. Data are expressed as ΔΔCt values relative to those in Tom^–^ cells from IR^wt/wt^/Gcre^Tg+^ control mice. *n* = 2–3 independent sorting by FACS (with each FACS sort containing pooled cells from 3 mouse stomachs of the same genotype). (**B**) Ghrelin levels in media from primary gastric mucosal cell cultures derived from ad libitum–fed control groups or GhIRKO mice treated for 6 hours with or without 1 nM insulin. *n* = 6–9 wells each. Data are presented as mean ± SEM. Data were calculated using repeated measures 2-way ANOVA, followed by a Sidak’s post hoc multiple-comparison test. *P < 0.05.

To selectively delete IRs from ghrelin cells, we crossed our validated Ghrelin-cre mouse line (27, 28, 45) with the Kahn lab’s conditional IR KO (IR^fl/fl^; B6.129S4[FVB]-*Insr^tm1Khn^*/J; The Jackson Laboratory) mouse line (46–50). Four experimental groups were generated, including ghrelin cell–selective insulin receptor–KO (GhIRKO) mice (IR^fl/fl^/Gcre^Tg+^ mice, with ghrelin cell–selective IR deletion) and 3 control genotypes (WT mice, IR^wt/wt^/Gcre^Tg–^; cre recombinase mice, IR^wt/wt^/Gcre^Tg+^; and floxed-IR mice, IR^fl/fl^/Gcre^Tg–^). We compared IR mRNA levels (as determined by quantitative PCR [qPCR]) in FACS-purified gastric ghrelin cells obtained from GhIRKO mice with those from IR^wt/wt^/Gcre^Tg+^ control mice, after those mice had been placed on a Rosa26-lox-STOP-lox-tdTomato reporter (B6.Cg-Gt26Sor^tm14[CAG–tdTomato]Hze/J^; The Jackson Laboratory) (51) background. We also compared IR mRNA expression in FACS-separated gastric ghrelin cells from nonghrelin gastric mucosal cells. Just as had been observed previously in gastric ghrelin cells FACS-purified from ghrelin-hrGFP mice (40), in IR^wt/wt^/Gcre^Tg+^ control mice, gastric ghrelin cells (Tom^+^) were enriched for IR mRNA as compared with nonghrelin gastric mucosal cells (Tom^–^) (Figure 1A). However, while IR mRNA expression in nonghrelin gastric mucosal cells from GhIRKO mice was not different from that in nonghrelin gastric mucosal cells from IR^wt/wt^/Gcre^Tg+^ control mice, IR mRNA expression was barely detectable in gastric ghrelin cells from GhIRKO mice (Figure 1A). Thus, ghrelin cell–selective IR deletion was confirmed in GhIRKO mice.

### Effects of insulin administration on ghrelin secretion and blood glucose–related parameters in GhIRKO mice.

**Figure 2:**
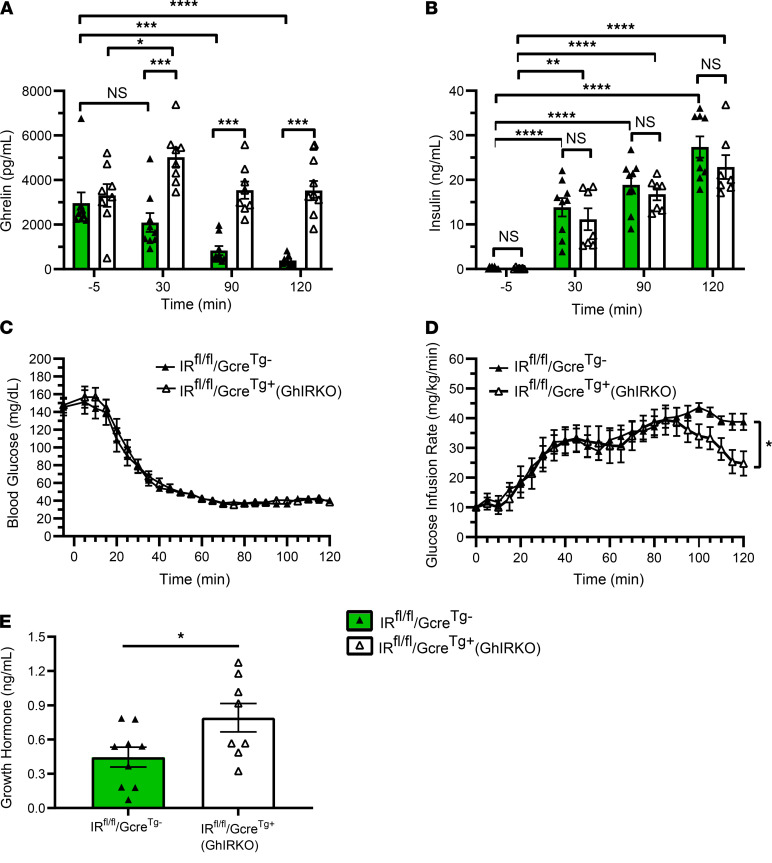
High plasma ghrelin in GhIRKO mice helps protect against insulin-induced hypoglycemia during hyperinsulinemic-hypoglycemia clamps. (**A**–**C**) Plasma ghrelin, plasma insulin, and blood glucose levels at various times during a 120-minute hyperinsulinemic-hypoglycemic clamp procedure. (**D**) Glucose infusion rates required to clamp the blood glucose levels within the target range (35–45 mg/dL) by the steady-state period (100–120 min). (**E**) Plasma GH at the end of clamps (t = 120). *n* = 8–10. Data are presented as mean ± SEM. Data for **E** were analyzed by 2-tailed Student’s *t* test. All other *P* values were calculated by repeated measures 2-way ANOVA, followed by Sidak’s post hoc multiple-comparison test. *P < 0.05, **P < 0.01, ***P < 0.001, ****P < 0.0001.

**Figure 3:**
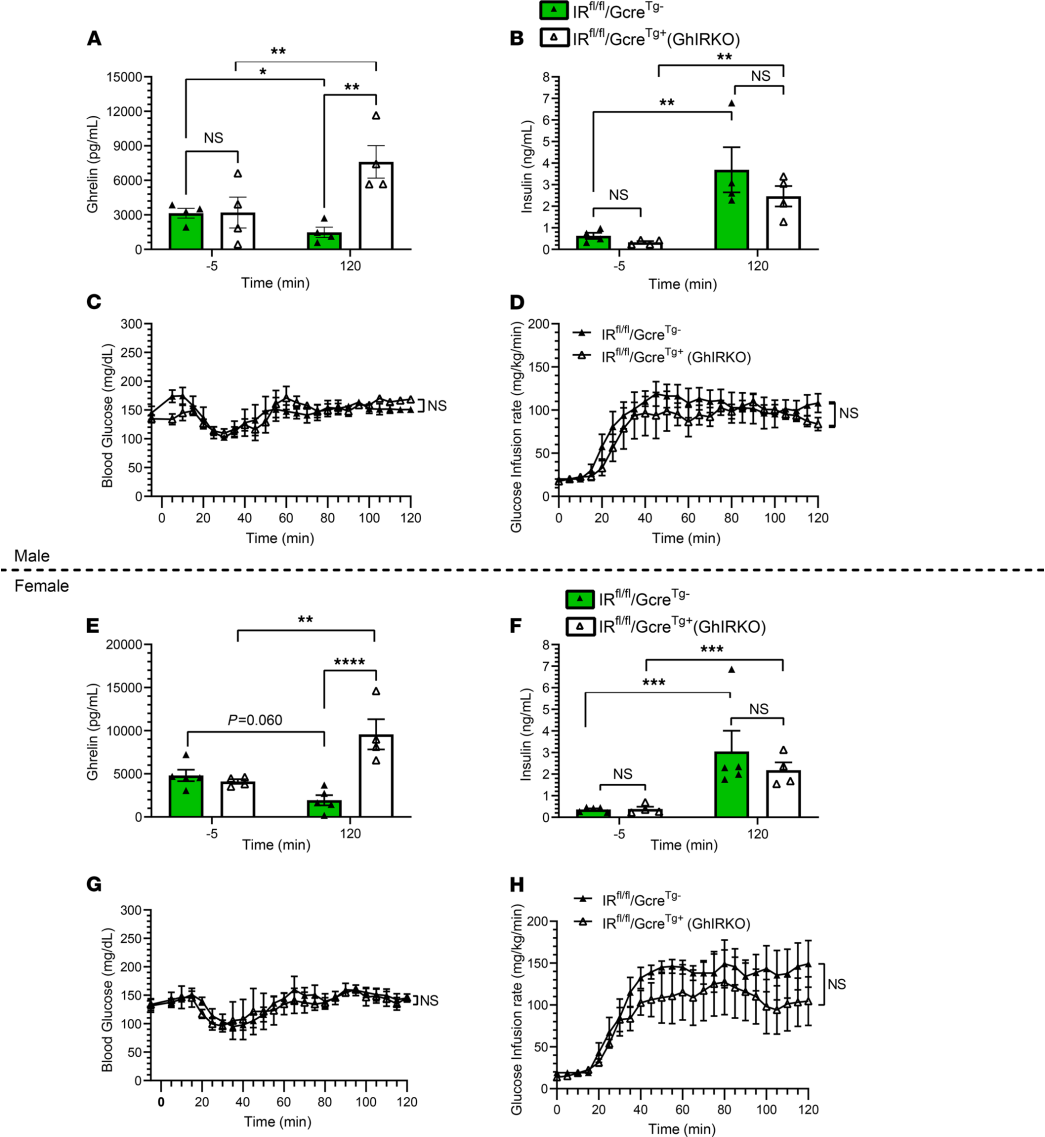
High insulin reduces plasma ghrelin during hyperinsulinemic-euglycemic clamps in WT mice but not in GhIRKO mice. (**A**–**H**) Data from hyperinsulinemic-euglycemic clamps performed in male (**A**–**D**) and female (**E**–**H**) mice. Plasma ghrelin (**A** and **E**), plasma insulin (**B** and **F**), and blood glucose levels (**C** and **G**) at various times during a 120-minute hyperinsulinemic-euglycemic clamp procedure. (**D** and **H**) Glucose infusion rates required to clamp the blood glucose levels within the euglycemic range (140–160 mg/dL) by the steady-state period (100–120 min). *n* = 4–5. Data are presented as mean ± SEM. All *P* values were calculated by repeated measures 2-way ANOVA, followed by Sidak’s post hoc multiple-comparison test. *P < 0.05, **P < 0.01, ***P < 0.001, ****P < 0.0001.

**Figure 4:**
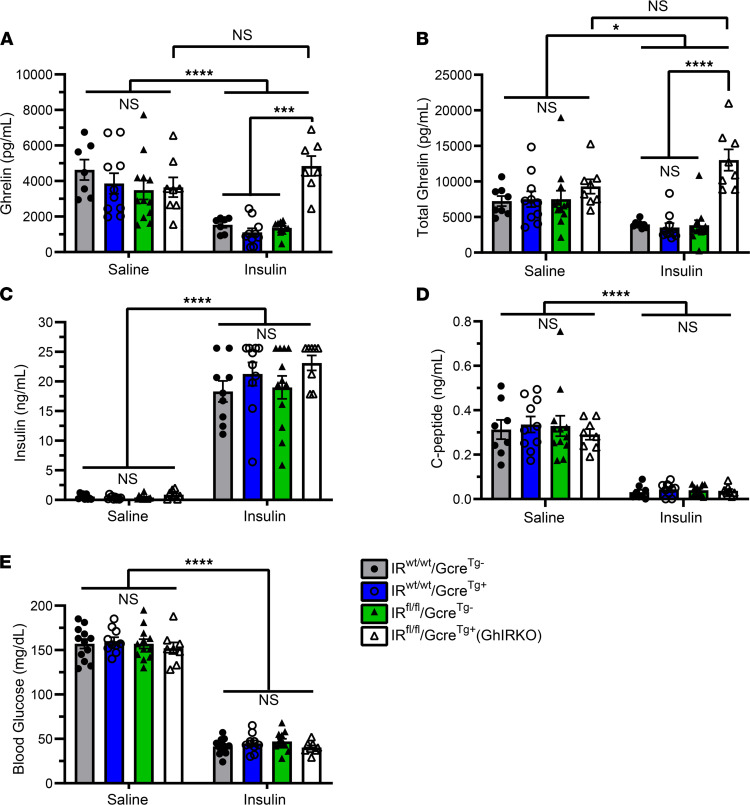
GhIRKO mice resist the actions of i.p. injected insulin to reduce plasma ghrelin during an acute insulin challenge. (**A** and **B**) Plasma ghrelin levels and plasma total ghrelin levels from GhIRKO mice and control littermates 30 minutes after i.p. insulin or saline administration. (**C**–**E**) Corresponding plasma levels of insulin, C-peptide, and blood glucose. *n* = 8–12. Data are presented as mean ± SEM. All *P* values were calculated using repeated measures 2-way ANOVA, followed by a Sidak’s post hoc multiple-comparison test. *P < 0.05, ***P < 0.001, ****P < 0.0001.

To test whether insulin reduces plasma ghrelin by directly suppressing ghrelin release from ghrelin cells, we first performed ex vivo ghrelin secretion studies using primary cultures of gastric mucosal cells isolated from GhIRKO and control mice (Figure 1B). Next, we performed hyperinsulinemic-hypoglycemic clamp studies (Figure 2) and hyperinsulinemic-euglycemic clamp studies on GhIRKO and control mice (Figure 3). Lastly, we performed an acute insulin challenge on GhIRKO and control mice (Figure 4). Of note, unless otherwise noted, the term “ghrelin” as used in this manuscript refers to acyl-ghrelin, whereas “total ghrelin” refers to acyl-ghrelin plus unacyl-ghrelin.

In the ex vivo preparation (Figure 1B), insulin (1 nM) suppressed secretion of ghrelin from gastric mucosal cells isolated from WT control mice, as had been shown previously (40). Insulin also suppressed ghrelin secretion from cells isolated from the 2 other control genotypes (IR^wt/wt^/Gcre^Tg+^ and IR^fl/fl^/Gcre^Tg–^). However, insulin had no effect on ghrelin secretion from cells isolated from GhIRKO mice.

Hyperinsulinemic-hypoglycemic clamps (20 mU insulin i.v./kg/min over 2 hours) induced a progressive decline in plasma ghrelin over the course of the 120-minute study in 8- to 10-week-old male IR^fl/fl^/Gcre^Tg–^ control mice but not in similarly aged GhIRKO mice (Figure 2A). Instead, at the 30-minute time point, ghrelin actually increased from its baseline level in GhIRKO mice. Plasma insulin levels (Figure 2B) and blood glucose levels (Figure 2C) achieved during the clamps were genotype independent. However, GhIRKO mice required a significantly lower glucose infusion rate to maintain blood glucose within the target hypoglycemic range during the final 20 minutes of the clamps (Figure 2D). Also, clamped GhIRKO mice exhibited significantly higher levels of the counterregulatory hormone GH than IR^fl/fl^/Gcre^Tg–^ controls (Figure 2E).

Since a low glucose environment can directly increase ghrelin release from primary cultures of gastric mucosal cells from WT mice (40), we next tested the effect of insulin administration using hyperinsulinemic-euglycemic clamps (4 mU insulin i.v./kg/min over 2 hours), which are not confounded by hypoglycemia. Similar to the above-described observations during the hyperinsulinemic-hypoglycemic clamps, plasma ghrelin levels fell over the course of the 120-minute hyperinsulinemic-euglycemic clamp study in 12- to 14-week-old male and female IR^fl/fl^/Gcre^Tg–^ control mice but not in similarly aged GhIRKO mice (Figure 3, A and E). Whereas plasma ghrelin decreased by ~56% in male IR^fl/fl^/Gcre^Tg–^ control mice (Figure 3A) and by ~59% in female IR^fl/fl^/Gcre^Tg–^ control mice (Figure 3E) at t = 120 minutes, plasma ghrelin increased by ~137% in male GhIRKO mice and by ~133% in female IR^fl/fl^/Gcre^Tg–^ control mice at t = 120 minutes compared with baseline (t = 0) (Figure 3, A and E). Plasma insulin levels (Figure 3, B and F) and blood glucose levels (Figure 3, C and G) at both baseline and t = 120 minutes were similar in GhIRKO and IR^fl/fl^/Gcre^Tg–^ control mice. Also, the glucose infusion rate needed to maintain blood glucose within the target euglycemic range was unaffected by genotype (Figure 3, D and H).

An acute insulin challenge (2.5 units of insulin i.p. per kg body weight) resulted in reductions in plasma ghrelin (Figure 4A) and plasma total ghrelin (which is a measure of both acyl-ghrelin and desacyl-ghrelin; Figure 4B) 30 minutes after i.p. insulin delivery in the 3 control genotypes but not in GhIRKO mice. Plasma insulin rose in all groups as a result of the administered insulin (Figure 4C). C-peptide reductions (Figure 4D) and blood glucose reductions (Figure 4E) were equivalent in all groups following insulin administration.

These findings using cultured gastric mucosal cells, hyperinsulinemic-hypoglycemic clamps, hyperinsulinemic-euglycemic clamps, and an acute insulin challenge confirm that ghrelin cell–expressed IRs mediate reductions in plasma ghrelin associated with administered insulin. The requirement for a lower glucose infusion rate in GhIRKO mice and their higher plasma GH levels during hyperinsulinemic-hypoglycemic clamps emphasize ghrelin’s key involvement in the counterregulatory response to insulin-induced hypoglycemia (24). The hyperinsulinemic-hypoglycemic clamp data further suggest that the usual direct effects of insulin on ghrelin cells to reduce ghrelin secretion in WT mice limits ghrelin’s full potential to protect against insulin-induced hypoglycemia. Also, the euglycemic clamp data suggest that ghrelin cell–selective IR deletion does not impact insulin sensitivity.

### Effects of food intake on plasma ghrelin in GhIRKO mice.

**Figure 5:**
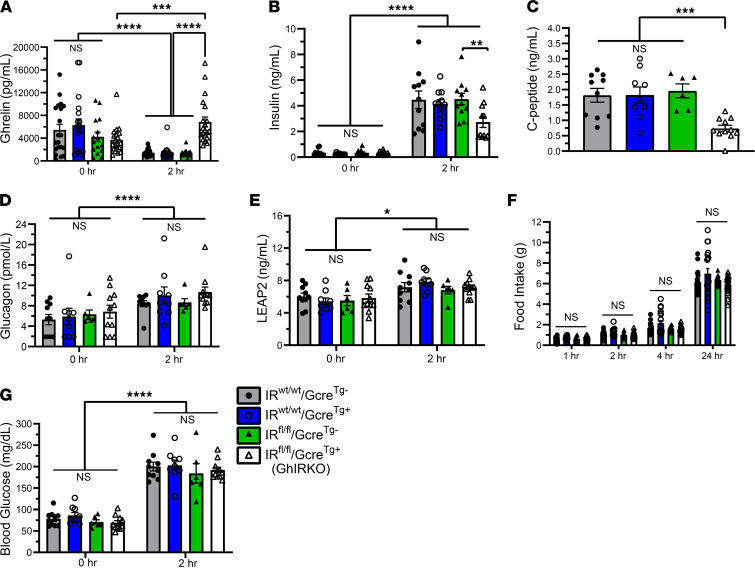
Refeeding following a 24-hour fast fails to suppress ghrelin secretion in GhIRKO mice. (**A**) Twenty-four–hour fasted plasma ghrelin levels (t = 0 hours) and plasma ghrelin levels after a 2-hour ad libitum refeeding period (t = 2 hours). (**B**) Plasma insulin (t = 0 hours and 2 hours). (**C**) Plasma C-peptide (t = 2 hours). (**D**) Plasma glucagon (t = 0 hours and 2 hours). (**E**) Plasma LEAP2 (t = 0 hours and 2 hours). (**F**) Rebound food intake measured at different time points following reintroduction of food at the end of the fast. (**G**) Blood glucose levels at the end of the 24-hour fast (t = 0 hours) and after the 2-hour refeeding period (t = 2 hours). *n* = 15–20. Data are presented as mean ± SEM. Data for **C** were analyzed by 1-way ANOVA, followed by Dunnett’s post hoc multiple-comparison test. All other *P* values were calculated by repeated-measures 2-way ANOVA, followed by Sidak’s post hoc multiple-comparison test. *P < 0.05, **P < 0.01, ***P < 0.001, ****P < 0.0001.

We used a 24-hour fast/refeed protocol to test whether meal-related declines in plasma ghrelin are dependent on insulin and its direct actions on ghrelin cell–expressed IRs (Figure 5). Two hours following reintroduction of standard chow diet to 24 hour–fasted mice, plasma ghrelin levels were significantly lower (by 67%–77%) than levels at the end of the fast (0 hours) in the 3 control genotypes (Figure 5A). In contrast, in GhIRKO mice, plasma ghrelin was significantly higher (by 85%) 2 hours following reintroduction of food than that at the end of the fast (Figure 5A). While 2-hour postprandial plasma insulin was higher than the end-of-fast levels in all genotypes, neither the postprandisal levels of insulin (Figure 5B) nor C-peptide (Figure 5C) were as high in GhIRKO mice as compared with the 3 control groups. The observed rises in plasma glucagon and plasma LEAP2 two hours following reintroduction of food were unaffected by ghrelin cell–selective IR deletion (Figure 5, D and E). Also, postprandial food intake (Figure 5F) and 2-hour postprandial blood glucose (Figure 5G) were genotype independent.

These results demonstrate that postprandial plasma ghrelin reductions result from engagement of ghrelin cell IRs by meal-related rises in insulin. Moreover, these results suggest the existence of a feedback loop between insulin and ghrelin in WT mice. In such a scenario, elevated insulin restricts ghrelin secretion, while the resulting decreased ghrelin further raises insulin via disinhibition of insulin secretion. Also in such a scenario, low insulin disinhibits ghrelin secretion, while the resulting increased ghrelin further lowers insulin via inhibition of insulin secretion. Such a feedback loop would explain the lower plasma insulin and C-peptide in GhIRKO mice following refeeding and presumably would result from an increase in the inhibitory tone normally provided by ghrelin to insulin-secreting pancreatic β cells either via GHSRs expressed by β cells and/or via somatostatin released from GHSR-expressing pancreatic δ cells (52–55). Notably, it is more likely that the attenuated rise in plasma insulin in GhIRKO mice results from the increased plasma ghrelin rather than vice versa, given that GhIRKO mice lack ghrelin cell–expressed IRs. Furthermore, it is unlikely that increased insulin sensitivity in refed GhIRKO mice would explain their pattern of an attenuated rise in insulin and C-peptide plus an equivalent rise in blood glucose (instead of an exaggerated rise blood glucose). Indeed, not only did the hyperinsulinemic-euglycemic clamp studies demonstrate equivalent insulin sensitivity in GhIRKO and IR^fl/fl^/Gcre^Tg–^ control mice (Figure 3, D and H), but previous studies have demonstrated that increased insulin sensitivity is associated with ghrelin or GHSR deletion (23, 24, 56, 57) — not with ghrelin overexpression.

### Effects of diet-induced obesity on plasma ghrelin in GhIRKO mice.

**Figure 6:**
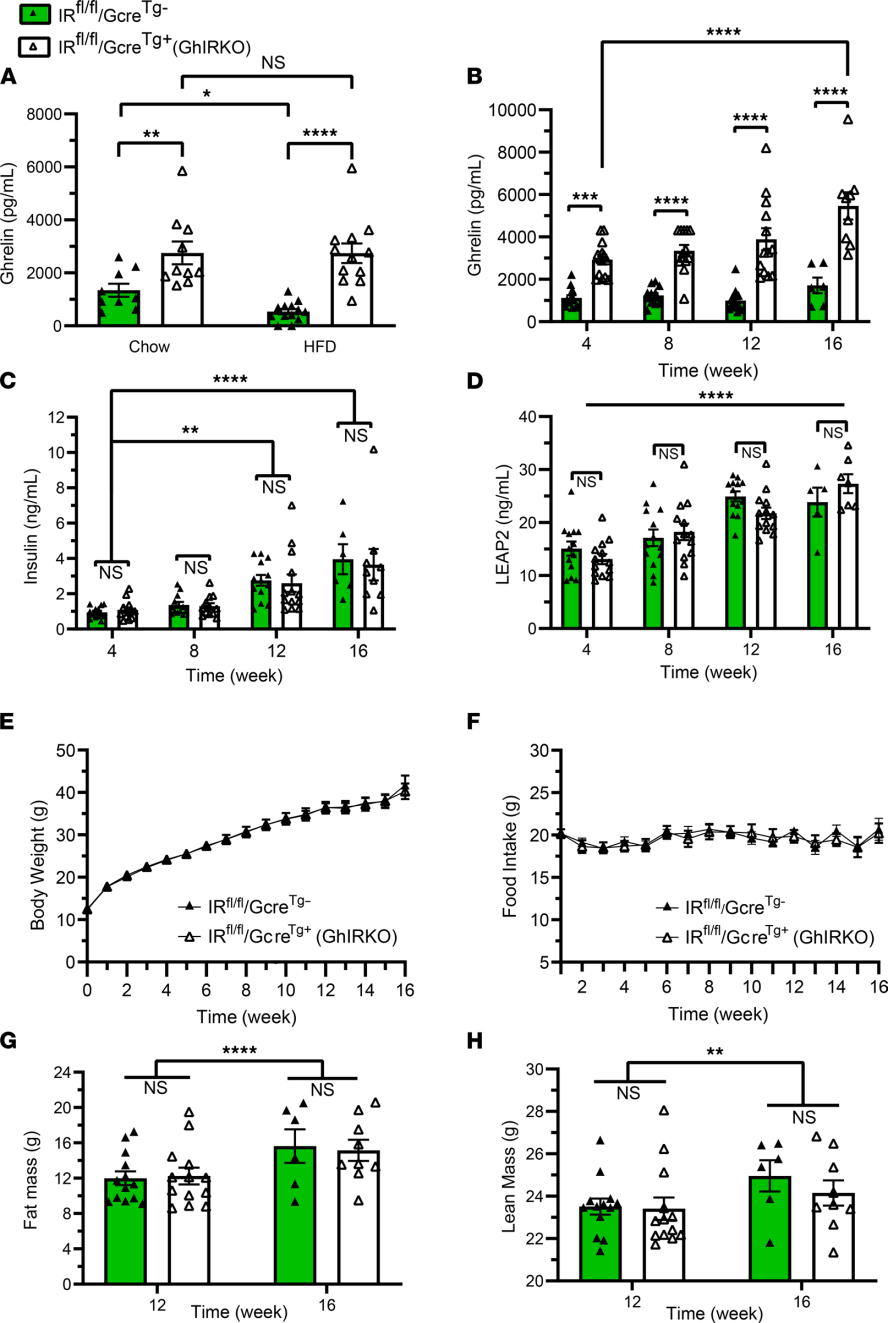
High-fat diet feeding fails to suppress plasma ghrelin in GhIRKO mice. (**A**) Plasma ghrelin levels of GhIRKO mice and IR^fl/fl^/Gcre^Tg–^ control mice fed standard chow or high-fat diet ad libitum for 8 weeks beginning at 4 weeks of age. *n* = 9–13. (**B**) Plasma ghrelin levels from high-fat diet–fed mice at various times during a 16-week–long high-fat diet challenge. (**C** and **D**) Corresponding plasma insulin levels and plasma LEAP2 levels. (**E** and **F**) Weekly body weights and weekly food intake. (**G** and **H**) Fat mass and lean mass as measured after 12 weeks and 16 weeks of high-fat diet. Data are presented as mean ± SEM. All *P* values were calculated by mixed-effects 2-way ANOVA, followed by Sidak’s post hoc multiple-comparison test. *P < 0.05, **P < 0.01, ***P < 0.001, ****P < 0.0001.

We provided individually housed GhIRKO mice and IR^fl/fl^/Gcre^Tg–^ control mice aged 4 weeks old ad libitum access to either standard chow or a 42% high-fat diet for 8 weeks. There was a genotype-independent effect of the 8 weeks of high-fat diet to increase body weights over those observed in standard chow–fed mice (IR^fl/fl^/Gcre^Tg–^: high-fat diet 30.5 ± 0.9 g versus standard chow 22.1 ± 0.4 g; GhIRKO: high-fat diet 30.8 ± 1.2 g versus standard chow 23.7 ± 0.5 g; *P* < 0.0001 for high-fat diet versus standard chow in both genotypes). Plasma ghrelin levels in IR^fl/fl^/Gcre^Tg–^ control mice fed a high-fat diet for 8 weeks were significantly lower than those observed in IR^fl/fl^/Gcre^Tg–^ mice fed standard chow, as expected (Figure 6A). Notably, plasma ghrelin levels in GhIRKO mice were higher than those in IR^fl/fl^/Gcre^Tg–^ mice, irrespective of diet. Furthermore, unlike that observed in IR^fl/fl^/Gcre^Tg–^ mice, high-fat diet–induced obesity was not associated with lower plasma ghrelin in GhIRKO mice.

The high-fat diet–fed GhIRKO mice and IR^fl/fl^/Gcre^Tg–^ control mice were continued on high-fat diet for another 8 weeks (Figure 6, B–H). As compared with high-fat diet–fed IR^fl/fl^/Gcre^Tg–^ control mice, plasma ghrelin in high-fat diet–fed GhIRKO littermates was significantly higher at all ages tested and also progressively rose over the 16-week study (Figure 6B). Insulin levels (Figure 6C) also progressively rose over the 16-week period, in a genotype-independent manner, as did plasma LEAP2 (Figure 6D). No differences were noted in body weight (Figure 6E), weekly food intake (Figure 6F), fat mass (Figure 6G), or lean mass (Figure 6H) between GhIRKO mice and IR^fl/fl^/Gcre^Tg–^ control mice.

These findings demonstrate that insulin acting via ghrelin cell–expressed IRs mediates obesity-associated declines in plasma ghrelin, while also limiting ghrelin secretion in standard chow–fed mice, albeit to a lesser degree (presumably, due to lower insulin levels in standard chow–fed versus high-fat diet–fed mice) (58). However, despite the elevated plasma ghrelin in high-fat diet–fed GhIRKO mice, body weight, food intake, and body composition were unaffected.

### Glucose gavage in GhIRKO mice.

**Figure 7:**
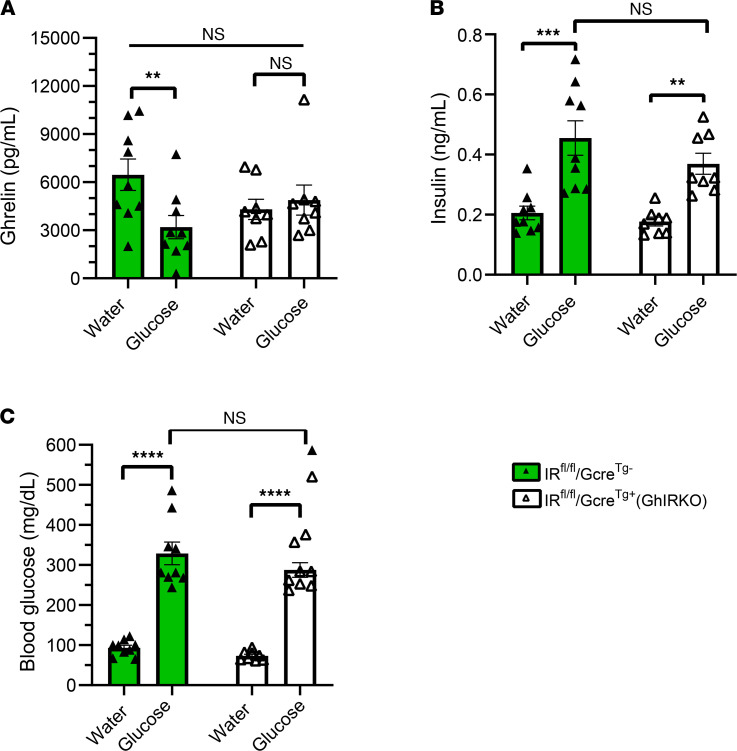
Glucose administration by gavage fails to suppress ghrelin levels in GhIRKO mice. (**A**) Plasma ghrelin levels 30 minutes following glucose versus water gavage of 24-hour–fasted mice. (**B** and **C**) Corresponding plasma insulin and blood glucose levels. *n* = 8–9. Data are presented as mean ± SEM. All *P* values were calculated by repeated-measures 2-way ANOVA followed by Sidak’s post hoc multiple-comparison test. **P < 0.01; ***P < 0.005; ****P < 0.001.

To determine whether the effects of glucose to reduce plasma ghrelin involve indirect mediation by insulin acting via ghrelin cell–expressed IRs, we administered glucose (2 g/kg body weight) by gavage to both GhIRKO and IR^fl/fl^/Gcre^Tg–^ control littermates (Figure 7). Glucose reduced plasma ghrelin in IR^fl/fl^/Gcre^Tg–^ control mice by ~50.5%, but it did not affect plasma ghrelin in GhIRKO mice (Figure 7A). The glucose gavage–induced increases in plasma insulin (Figure 7B) and blood glucose (Figure 7C) were genotype independent.

These results suggest that the effects of glucose to inhibit ghrelin secretion via direct actions on ghrelin cells previously demonstrated in cultured gastric mucosal cells (35, 40) do not contribute substantively to ghrelin secretion in vivo. Instead, glucose’s indirect actions to stimulate secretion of insulin, which in turn engages ghrelin cell–expressed IRs to inhibit ghrelin release, predominate in vivo.

## Discussion

**Figure 8:**
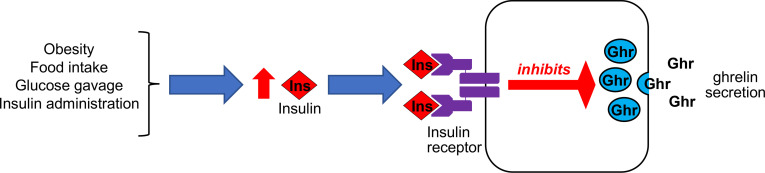
Proposed model by which ghrelin cell–expressed IRs act to control ghrelin secretion in various metabolic settings. In obesity, the postprandial state, and following glucose gavage, high plasma insulin levels engage ghrelin cell–expressed IRs to inhibit ghrelin secretion. The same occurs upon insulin administration.

The data herein demonstrate that increased insulin, acting via ghrelin cell–expressed IRs to reduce ghrelin secretion, is required for postprandial and obesity-associated reductions in plasma ghrelin. Ghrelin cell–expressed IRs also mediate the reductions in plasma ghrelin resulting from insulin administration. A proposed model by which ghrelin cell–expressed IRs act to control ghrelin secretion in the metabolic settings tested here (diet-induced obesity, postprandial state, glucose gavage, and upon insulin administration) is presented schematically in Figure 8.

The large impact of this indirect, insulin-mediated mechanism to suppress ghrelin secretion in postprandial and obesity settings is somewhat surprising, especially since direct mechanisms exist for circulating macronutrients (such as those that would be derived from meals or as a result of diet-induced obesity) to suppress ghrelin secretion. Ghrelin cells are enriched for the fatty acid receptors GPR43 and GPR120 — as well as the amino acid taste receptors CaSR, GPRC6A, and TAS1R1-TASR1R3 — and their activation reduces ghrelin secretion when tested ex vivo or using ghrelinoma cell lines (28–31, 33). Also, glucose can directly be taken up and metabolized by ghrelin cells, leading to suppressed ghrelin release (35, 40). Perhaps the previous observation in cultured gastric mucosal cells that raising the glucose concentration in the cell culture medium from 5 mM (representative of normoglycemia) to 10 mM (representative of hyperglycemia) reduces ghrelin release only minimally, whereas lowering it from 5 mM to 1 mM (representative of hypoglycemia) or to 0 mM enhances ghrelin release, alludes to a minimal role for a direct inhibitory effect of glucose on ghrelin secretion in vivo (35). Certainly, the current study demonstrates that orally administered glucose had no effect to reduce plasma ghrelin in GhIRKO mice. Also of interest, previous work revealed that glucose’s direct effects to inhibit ghrelin secretion from cultured gastric mucosal cells are lacking in cells taken from diet-induced obese mice (3). That observation supports the current findings, suggesting a major role for insulin and ghrelin cell–expressed IRs in regulating ghrelin secretion in the setting of diet-induced obesity. Future studies in GhIRKO mice could help determine whether orally administered lipids or proteins also are reliant on ghrelin cell–expressed IRs for their inhibitory effects on ghrelin release versus whether their direct effects on ghrelin release also figure prominently. Furthermore, while the present findings confirm that meal- and obesity-associated plasma ghrelin reductions require ghrelin cell–expressed IRs, future studies could help determine the mechanisms responsible for the rises in plasma ghrelin observed in GhIRKO mice 2 hours after the reintroduction of food following a 24-hour fast (Figure 5A), 30 minutes after starting the hyperinsulinemic-hypoglycemic clamp procedure (Figure 2A), 120 minutes after starting the hyperinsulinemic-euglycemic clamp procedure (Figure 3, A and E), or after chronic exposure to high-fat diet (Figure 6B). For instance, might the known stimulatory effects of the sympathoadrenal system and/or low glucose on ghrelin cells run unopposed to increase ghrelin secretion in one or more of these scenarios without the actions of ghrelin cell–expressed IRs (27, 35, 40)?

Also worthy of discussion, while the elevated plasma ghrelin observed in GhIRKO mice affected many key metabolic parameters, not all parameters tested were impacted. As such, in the hyperinsulinemic-hypoglycemic clamp studies, GhIRKO mice required lower glucose infusion rates and had higher plasma GH. This observation confirms previous work indicating that ghrelin helps protect against insulin-induced hypoglycemia and that this effect may involve its actions as a GH secretagogue (24). Also, in the fast/refeed study, GhIRKO mice exhibited lower plasma insulin and C-peptide levels. This finding emphasizes the capacity of the elevated ghrelin to feed back onto pancreatic islets to restrict insulin release (54, 55). However, GhIRKO mice and control mice exhibited similar blood glucose and plasma C-peptide levels in the acute insulin challenge, similar food intake and blood glucose responses in the fast/refeed study, and similar body weights, food intake, body composition, blood glucose, and plasma insulin in the diet-induced obesity model. Also, GhIRKO mice and control mice required similar glucose infusion rates during the hyperinsulinemic-euglycemic clamp procedure, suggesting unchanged insulin sensitivity. These differential responses to the elevated ghrelin may be related to variability in the degree to which the various mediators of ghrelin action succumb to ghrelin resistance in different metabolic settings (6). For instance, obesity is regarded as a state of ghrelin resistance, as evidenced by the failure of administered ghrelin to acutely induce food intake in diet-induced obese mice and in obese agouti mice (59–61), the failure of administered ghrelin to reduce energy expenditure in diet-induced obese mice (62), and the attenuated ghrelin-induced GH release in human subjects with obesity (63). Among the pathways proposed to mediate the ghrelin resistance of obesity is a coordinated rise in plasma LEAP2 and a fall in plasma ghrelin observed in both humans and mice with obesity (64). Indeed, based on the relative plasma levels of LEAP2 and ghrelin in both obese states and in fed states, and their similar binding affinities for GHSR, it has been proposed that LEAP2 serves as the dominant ligand of GHSR, prominently antagonizing ghrelin actions, in both obese and fed states (64). However, here — despite the elevation of ghrelin observed in GhIRKO mice in the long-term high-fat diet study — LEAP2 levels remained just as high as those in control mice. Perhaps this persistently high LEAP2 was still able to prevent the elevated ghrelin in GhIRKO mice from exerting its metabolic effect in the setting of diet-induced obesity. Neutralizing plasma LEAP2, which has been shown previously to increase fasting GH levels (16), might help us in future studies to resolve the mechanism underlying the observed varying physiological effects of elevated ghrelin in GhIRKO mice. Notwithstanding these differing phenotypic effects of the elevated ghrelin, the current data may have implications for ghrelin-targeted therapies aimed at treating individuals susceptible to hypoglycemia. For instance, therapies that could achieve ghrelin cell–selective IR blockade would presumably help protect against insulin-induced hypoglycemia (by preventing insulin-induced drops in ghrelin) without gains in body weight, which are often considered unwelcome.

## Methods

### Animal care.

Mice were housed at 21.5°C–22.5°C with a 12-hour light/12-hour dark cycle with free access to standard chow (2916, Teklad Global 16% Protein Rodent Diet, Envigo) and water, unless otherwise noted. Body weights of chow-fed mice were determined at the start of each of the metabolic manipulations performed and were found to be equivalent between genotypes. All experiments were approved by the UT Southwestern Medical Center Institutional Animal Care and Use Committee.

### Generation of mice with ghrelin cell–selective IR deletion.

Ghrelin-cre mice (27, 28) on a C57BL/6N genetic background were crossed with heterozygous IR^fl/wt^ mice (46) (B6.129S4[FVB]-Insrtm1Khn/J; stock: 006955, The Jackson Laboratory) to generate breeders harboring one copy of the loxP-flanked IR allele and one copy of the Ghrelin-cre Tg (Gcre^Tg+^). These mice were bred with heterozygous IR^fl/wt^ mice to generate the 4 study groups, which included mice containing 2 loxP-flanked IR alleles or 2 WT IR alleles, all with or without 1 copy of the Ghrelin-cre Tg.

### Generation of mice with ghrelin cell–selective IR deletion on a ghrelin cell tdTomato reporter background.

Ghrelin-cre mice were bred to Rosa26-lox-STOP-lox-tdTomato mice (B6.Cg-Gt26Sortm14[CAG–tdTomato]Hze/J; stock: 007914, The Jackson Laboratory) to generate Ghrelin-cre–tdTomato reporter mice, which in turn were bred to IR^fl/wt^ mice to generate IR^fl/wt^/Gcre^Tg+^ mice and IR^fl/wt^/tdTomato mice. Next, the IR^fl/wt^/Gcre^Tg+^ mice were crossed with IR^fl/wt^/tdTomato mice to generate IR^fl/fl^/Gcre^Tg+^/tdTomato mice (GhIRKO mice expressing tdTomato selectively in ghrelin cells) and IR^wt/wt^/Gcre^Tg+^/tdTomato mice (Ghrelin-cre control mice expressing tdTomato selectively in ghrelin cells). Gastric ghrelin cells (with tdTomato, Tom^+^) and nonghrelin gastric cells (without tdTomato, Tom^–^) from these 2 genotypes were FACS purified. IR mRNA levels were quantified by qPCR to validate ghrelin cell–selective deletion of the IR gene only in IR^fl/fl^/Gcre^Tg+^/tdTomato mice but not in IR^wt/wt^/Gcre^Tg+^/tdTomato control mice. Procedures for FACS and qPCR are described below.

### Acute insulin challenge.

Eight- to 10-week-old male mice were singly housed to acclimatize for 7 days before experimentation. Beginning at 7:00 a.m. on the day of experiment, mice were fasted for 3 hours before testing. Fasting blood glucose (t = –5 minutes) was measured from nicked tails using a Contour Next EZ monitoring system (Bayer). Insulin (Humulin-R; Lilly) was diluted in sterile saline (0.9%) and injected at 2.5 units/kg body weight i.p. at t = 0 minutes. Saline (0.9%) served as the vehicle control. Blood glucose was measured from nicked tails again at 30 minutes after injection. Blood was collected from the superficial temporal vein at 30 minutes for plasma ghrelin, total ghrelin, insulin, and C-peptide determinations.

### FACS.

A mix of male and female GhIRKO or control littermate mice (*n* = 6–9) were deeply anesthetized with chloral hydrate (700 mg/kg i.p.), followed by laparotomy. The stomachs were tied off at both ends with surgical sutures (nylon 5-0), excised, placed in ice-cold PBS, and then incised at the nonglandular portion to clear the digesta. The stomachs were inverted inside-out by passing the distal part of the stomach through the incision; they were then inflated with and briefly placed in ice-cold DMEM medium lacking glucose (Invitrogen) until all stomachs in the group were isolated. The residual digesta adhering to the stomach mucosa was cleared off gently by using soft paper pads. The stomachs were then incubated for 1.5 hours at 37°C in 35 U dispase II/3 mL PBS (Roche Diagnostics) in a 50 mL centrifuge tube; then, the mucosa was scraped off using a transfer pipette into sterile DMEM/F-12 (1:1) medium (Mediatech Inc.) containing 10% (vol/vol) FBS (Atlanta Biologicals), supplemented with 100 U/mL penicillin and 100 μg/mL streptomycin sulfate. The cells were centrifuged at 300*g* for 3 minutes, and the media was removed and subjected to digestion with 0.25% trypsin EDTA (Mediatech Inc.). Trypsin was inactivated after 5 minutes with addition of FBS containing DMEM/F-12 medium, and the mucosal cells were brought up and down into a plastic transfer pipette to disperse them. The suspension was then passed through a 100 μM filter and then centrifuged at 310*g* for 3 minutes to remove the supernatant medium. The resulting cell pellets were suspended in 1 mL FACS buffer (3% FBS, 0.5 mM EDTA, 0.1% BSA, and 10 U/mL DNase I) and passed through a 30 μM filter.

The isolated gastric mucosal cells (from 3 stomachs per FACS) were next subjected to FACS analysis using a BD FACSAria III Cell sorter and analyzed using BD FACSDiva 8.0.2 software at the UT Southwestern Flow Cytometry Core to separate enriched populations of Tom^+^ ghrelin cells, as well as Tom^–^ nonghrelin gastric mucosal cells. The cells were collected directly into buffer provided with the Arcturus PicoPure RNA Isolation Kit (Applied Biosystems, Thermo Fisher Scientific) and were stored at –80°C until qPCR analysis. Cells from 2–3 independent FACS sorts (each sort containing cells pooled from 3 mouse stomachs of the same genotype) were used for qPCR analysis.

### Primary gastric mucosal cell culture.

Primary gastric mucosal cells were isolated from 3 stomachs of the same genotype and dispersed enzymatically and mechanically as described above for FACS analysis, with the following changes. For primary cultures, the cells were suspended in FBS containing DMEM/F-12 medium instead of FACS buffer and plated at a density of 1 × 10^5^ cells/mL/well supplemented with sodium octanoate–BSA to achieve a final concentration of 50 μM in poly-D-lysine precoated 24-well plates. The cells were placed overnight in a 37°C incubator with 5% CO_2_. The next day, the cells were treated with 1 nM insulin (Sigma-Aldrich) or vehicle (saline) for 6 hours in serum free DMEM (Invitrogen) containing 5 mM glucose and 50 μM sodium octanoate–BSA. Three replicates (*n* = 3) were run for each treatment (insulin versus vehicle) and for each genotype. At the end of the 6-hour incubation period, the medium was collected, placed on ice, and immediately centrifuged at 4°C at 800*g* for 5 minutes. Hydrochloric acid was added to the supernatant to achieve a final concentration of 0.1N (for stabilization of acyl-ghrelin) and stored at –80°C until analysis.

### Fast/refeeding.

Eight- to 10-week-old female mice were singly housed and gently handled 3 days before performing the experiment. Mice were fasted for 24 hours, starting at 9:00 a.m. The next day at 9:00 a.m., preweighed standard chow diet was provided ad libitum for 2 hours at the bottom of the cage. After 2 hours, the remaining chow diet was reweighed to calculate the consumed diet. One week later, this fast/refeeding experiment was repeated to measure blood glucose and hormone levels in the same mice. Blood glucose was measured at t = 0 and t = 120 minutes using nicked tails. Also, blood was collected from nicked tails at t = 0 to measure plasma ghrelin, insulin, glucagon, and LEAP2. Following 2-hour ad libitum access to standard chow diet, blood was again collected from nicked tails at t = 120 minutes to measure plasma ghrelin, insulin, glucagon, and LEAP2. Blood for plasma C-peptide measurement was collected by quick superficial temporal vein bleed at t = 120 minutes.

### Hyperinsulinemic-hypoglycemic clamps.

Eight- to 10-week-old male mice were anesthetized using 2% isoflurane, and each was then surgically implanted with a right jugular vein catheter (0.20-inch, Silastic tubing, Instech Laboratories). The free end of the catheter was exteriorized from the dorsal intrascapular region, and the incision sites were closed with a 5-0 nylon suture. Mice were fitted with a vascular harness (Instech Laboratories). Mice were provided carprofen (5 mg/kg, s.c.) immediately, 24 hours, and 48 hours after surgery for analgesia. Mice also were closely postsurgically monitored for signs of infection or swelling, neither of which were observed.

Hyperinsulinemic-hypoglycemic clamps were performed in conscious, unrestrained mice as previously described with few modifications (24). Briefly, 5 days after jugular vein catheterization, mice were fasted for 5 hours (starting at 8:00 a.m., with access to water until 12:30 p.m.). Food and water were restricted during the clamp procedure. Insulin was infused at 20 mU/kg/min i.v. over 2 hours. A 50% glucose solution was simultaneously infused i.v. at a variable rate to achieve hypoglycemia in the range of 35–45 mg/dL during the final 20 minutes (steady-state period). Blood glucose was measured via tail nicks every 5 minutes. Blood samples to measure ghrelin and insulin were taken from tail nicks at t = –5, 30, 90, and 120 minutes. At 120 minutes, additional blood samples were collected by quick decapitation to measure GH.

### Hyperinsulinemic-euglycemic clamps.

Hyperinsulinemic-euglycemic clamps were performed in a separate set of 12- to 14-week-old male and female mice by following the above hyperinsulinemic-hypoglycemic clamp procedure, with the following modifications: insulin was infused at 4 mU/kg/min i.v. over 2 hours. A 50% glucose solution was simultaneously infused i.v. at a variable rate to achieve euglycemia in the range of 140–160 mg/dL during the final 20 minutes. Blood samples to measure ghrelin and insulin were taken from tail nicks at t = −5 and 120 minutes. GH was not measured.

### Long-term feeding studies.

Four-week-old male GhIRKO and IR^fl/fl^/Gcre^Tg–^ littermates were individually housed and fed high-fat diet (Envigo Teklad TD88137; with an energy density of 4.5 kcal/g, of which 42% of kcal are derived from fat) ad libitum for 16 weeks. Body weight and food intake were measured weekly. Body composition analysis was performed using an EchoMRI-100 apparatus (EchoMRI LLC) after 12 weeks and 16 weeks of exposure to high-fat diet. Blood was collected from nicked tails of ad libitum–fed mice into EDTA-coated microtubes after 4, 8, 12, and 16 weeks of high-fat diet feeding to measure plasma ghrelin, total ghrelin, insulin, and LEAP2. Notably, the plasma ghrelin levels of GhIRKO and IR^fl/fl^/Gcre^Tg–^ mice after 8 weeks of high-fat diet feeding were determined twice from the same samples: the first time, the 8-week plasma samples were run at the same time as the 4-week, 12-week, and 16-week plasma samples (Figure 6B). The second time, the remaining available 8-week plasma samples were run at the same time as plasma samples taken from male GhIRKO and IR^fl/fl^/Gcre^Tg–^ mice that had been fed standard chow diet beginning at 4 weeks of age for 8 weeks (Figure 6A).

### Glucose gavage.

Male mice (10–12 weeks old) were singly housed for 7 days prior to experimentation. One day before the experiment, mice were fasted for 24 hours starting at 9 a.m. The next day, glucose (Sigma-Aldrich) 2 g/kg was dissolved in water and administered by oral gavage. Water served as a vehicle control. Blood was collected at 30 minutes after gavage from the superficial temporal vein for measurement of ghrelin and insulin. Blood glucose was also measured using a handheld Contour Next EZ monitoring system from the blood collected from the superficial temporal vein.

### Blood collection and determination of plasma hormone levels.

Blood samples were collected by quick superficial temporal vein bleed, tail nicks, or following quick decapitation as specified into EDTA-coated microtubes kept on ice. The collection tubes contained either the protease inhibitors p-hydroxymercuribenzoic acid (Sigma-Aldrich; final concentration 1 mM; for ghrelin measurement) or aprotinin (Sigma-Aldrich; final concentration 250 KIU/mL; for glucagon and LEAP2 measurement) or no additional protease inhibitor (for insulin, C-peptide, and GH measurements). The samples were immediately centrifuged at 4°C at 1500*g* for 15 minutes. For acyl-ghrelin stabilization, HCl was added (1:10) to the p-hydroxymercuribenzoic acid–treated plasma to achieve a final concentration of 0.1N. Processed samples were stored at –80°C in small aliquots until analysis of plasma hormone levels.

ELISA kits were used for ghrelin, total ghrelin, and GH (MilliporeSigma), insulin and C-peptide (Crystal Chem), glucagon (Mercodia AB), and LEAP2 (Phoenix Pharmaceuticals Inc.). Calorimetric assays were performed using a BioTek PowerWave XS Microplate spectrophotometer (BioTek) and BioTek KC4 junior software.

### qPCR.

Total RNA was isolated from sorted cells using the guanidinium thiocyanate-phenol/chloroform extraction method by addition of RNA STAT-60 or using the Arcurus PicoPure RNA Isolation Kit (Thermo Fisher Scientific). The isolated RNA was quantified using a Nanodrop spectrophotometer (Thermo Fisher Scientific). All of the total RNA that was isolated from the sorted cells was treated with ribonuclease-free deoxyribonuclease (Roche), and complementary DNA was synthesized by reverse transcription using SuperScript III (Invitrogen). qPCR was performed using the QuantStudio 5 System (Thermo Fisher Scientific) and a combination of TaqMan primer/probe chemistry for 18s rRNA (Mm04277571_s1) and IR (Mm01211881_m1) or SYBR chemistry for tdTomato (forward primer, 5′-CACCATCGTGGAACAGTACGA-3′; reverse primer, 5′-GCCATGCCCCAGGAACA-3′). The reaction mixtures for qPCR contained 2.0 μL reverse-transcribed RNA, 1 μL of the assay on demand, and 5 μL of 2× TaqMan Gene Expression master mix (Thermo Fisher Scientific). The IR mRNA levels are represented relative to 18s rRNA (the invariant control gene), as calculated by the comparative threshold cycle (ΔΔCt) method, and in turn, relative to the ΔΔCt values in Tom^–^ cells in IR^wt/wt^/Gcre^Tg+^ controls.

### Statistics.

Data are presented as mean ± SEM. Data were analyzed by 2-tailed unpaired Student’s *t* test, 1-way ANOVA, or 2-way ANOVA followed by post hoc comparison tests, as indicated in the figure legends. All data were analyzed using GraphPad prism version 9.0.0. Outliers, if any, were removed using Rout test. *P* values less than 0.05 were considered statistically significant.

## Author contributions

KS, BKM, and JMZ developed the concept. KS, EDB, and JMZ developed the experimental strategy. KS, ST, DG, SV, SOL, and NPM performed the experiments. CPR worked to organize the breeding and all aspects of animal husbandry required for the euglycemic clamp studies. KS, ST, and JMZ analyzed the data. EDB and JMZ secured funding for the project. BKM helped edit the paper. KS and JMZ wrote the paper.

## Version 1 09/02/2021

In-Press Preview

## Version 2 09/22/2021

Electronic publication
